# Cone dystrophy associated with autoimmune polyglandular syndrome type 1

**DOI:** 10.1038/s41598-023-38419-9

**Published:** 2023-07-11

**Authors:** Abdulrahman Badawi, Moustafa Magliyah, Omar Alabbasi, Lama AlAbdi, Fowzan S. Alkuraya, Patrik Schatz, Hani Basher ALBalawi, Marco Mura

**Affiliations:** 1 Vitreoretinal Division, King Khaled Eye Specialist Hospital, Riyadh, Saudi Arabia; 2 Ophthalmology Department, Prince Mohammed Medical City, AlJouf, Saudi Arabia; 3 Ophthalmology Department, Almadinah Almonawwarah Hospital, Madinah, Saudi Arabia; 4 Department of Translational Genomics, Center for Genomic Medicine, King Faisal Specialist Hospital and Research Center, Riyadh, Saudi Arabia; 5 Department of Zoology, Collage of Science, King Saud University, Riyadh, Saudi Arabia; 6 Department of Anatomy and Cell Biology, College of Medicine, Alfaisal University, Riyadh, Saudi Arabia; 7 Department of Ophthalmology, Clinical Sciences, Skane University Hospital, University of Lund, Lund, Sweden; 8 Ophthalmology Division, Department of Surgery, Faculty of Medicine, University of Tabuk, Tabuk City, Saudi Arabia

**Keywords:** Genetics, Molecular biology, Neuroscience, Molecular medicine, Pathogenesis, Endocrine system and metabolic diseases, Eye diseases

## Abstract

To report the association of autoimmune polyglandular syndrome type 1 (APS1) with cone dystrophy in a large Saudi family. This is a Retrospective chart review and prospective genetic testing and ophthalmic examination of a large multiplex consanguineous family. Genetic testing was performed on 14 family members, seven of whom had detailed ophthalmic examinations. Medical history, ocular history and evaluation, visual field testing, full-field electroretinogram (ERG), and Whole Exome Sequencing (WES) results were analyzed. Three family members were homozygous for c.205_208dupCAGG;p.(Asp70Alafs*148) in *AIRE* and homozygous for c.481-1G>A in *PDE6C*. One additional family member was homozygous for only the *AIRE* variant and another additional family member was homozygous for only the *PDE6C* variant. All patients with homozygosity for the *PDE6C* variant had cone dystrophy, and all patients with homozygosity for the *AIRE* variant had APS1. In addition, two of the family members who were homozygous for the *PDE6C* and *AIRE* variants had reduced rod function on ERG. We report the co-inheritance for *APS1* and *PDE6C*-related cone dystrophy, an unusual example of two seemingly independent recessive conditions coinciding within a family. Dual molecular diagnosis must be taken into account by ophthalmologists facing unusual constellations of findings, especially in consanguineous families.

## Introduction

Autoimmune Polyglandular Syndrome type1 (APS1; OMIM 240300) is a subtype of Autoimmune Polyendocrine Syndromes (APS) which are autoimmune disorders involving several endocrine organs. APS1 is characterized by a triad of Addison’s disease, hypoparathyroidism and mucocutaneous candidiasis among several other endocrine and nonendocrine manifestations^1^. It is associated with biallelic (recessive) or monoallelic (dominant) pathogenic variants in the human autoimmune regulatory (*AIRE*) gene^2^, which encodes a 545-amino-acid proline-rich (APECED) protein with a molecular weight of 58 kD. APECED protein is mainly localized to the cell nucleus, where it plays a role in regulating genetic transcription in normal cells^3^. Pathogenic variants in *AIRE* gene lead to changes in intracellular localization of APECED protein, altering its role in transcription^4^.

Ocular manifestations of APS1 include autoimmune keratopathy, dry eyes, and pigmentary retinopathy^5^. The mechanisms for the development of ocular surface disease in patients with APS1 are not completely understood. APECED is thought to regulate the expression of non-thymic proteins within the thymus to facilitate the elimination of self-reactive T cells^6^. APECED deficiency leads to infiltration of CD4+ and CD8+ T cells on the ocular surface and meibomian glands of *Aire*-mutant mice^6^. An increased expression of proinflammatory cytokines by ocular surface cells was also seen^7^. Therefore, immune-mediated mechanisms may play a role in the loss of ocular surface barrier function, decreased goblet cell density and increased epithelial stratification^7^. These factors, in turn, lead to severe blepharitis and keratoconjunctivitis in patients with APS1.

In addition, a target eye antigen [interphotoreceptor retinoid-binding protein (IRBP)] was found to be a dominant eye autoantigen in *Aire*-mutant mice^8^. This may underlie a possibly autoimmune retinopathy in patients with APS1.

APS1 has not been previously associated with cone dystrophy. Here we report for the first time the association of APS1 with *PDE6C*-related cone dystrophy, in a large inbred Saudi family. This is a rare co-occurrence of two homozygously inherited recessive conditions. We discuss the clinical implications of this phenomenon in the practice of ophthalmology especially in highly consanguineous populations.

## Methods

This study was conducted at King Khaled Eye Specialist Hospital (KKESH), according to the tenets of the Declaration of Helsinki, and approved by the Institutional Review Board at KKESH (Project 22088-R). Consent for genetic testing and participation in this study was obtained from all participants (KFSHRC RAC#2070 023). Rare (allele frequency ≤ 0.001) homozygous and compound heterozygous loss of function variants or variants with damaging in silico predictions that lie within the coding or splicing regions of protein-coding genes were considered. After identification of the index case through discovering pathogenic variants in *AIRE* and *PDE6C* genes, all available family members were recruited for further genetic testing. Due to the lack of ophthalmic facilities which allow for proper ophthalmic evaluation of the family members in their home city, prospective ophthalmic examination was performed on 7 available family members to document the ophthalmic findings. Collected data included age, gender, past medical and family history including parental consanguinity; best-corrected visual acuity (VA), refractometry, slit-lamp biomicroscopy, and fundus examination; full-field electroretinography (ERG) according to the institution’s protocol previously reported^9^; multimodal imaging including macular spectral-domain optical coherence tomography (SD-OCT, Spectralis OCT, Heidelberg Engineering, Inc., Heidelberg, Germany), color fundus photos (Topcon TRC-50DX, Topcon Medical Systems, Inc., NJ, US), ultra-widefield pseudocolor fundus photos and fundus autofluorescence (FAF) (Optos PLC, Dunfermline, UK). Genetic analysis was performed using whole exome sequencing in the index case and targeted variant analysis by Sanger sequencing in the other family members. Both vaiants in *AIRE* and *PDE6C* genes which have led to the diagnosis of APS1 and cone dystrophy, respectively in this paper were described previously by some of the authors^10^.

### Ethical approval

Ethical approval was obtained from the Institutional Review Board (IRB) at King Khaled Eye Specialist Hospital (KKESH) with approval ID: Project 22088-R. Written informed consents to participate in this study and for their clinical details to be known were obtained from all patients and legal guardians of participants below 16 years. This study was conducted in compliance with the guidelines of the Declaration of Helsinki.

### Informed consent

Written informed consents for publication of this study, the details and accompanying images were obtained from all involved patients.

## Results

**Figure 1:**
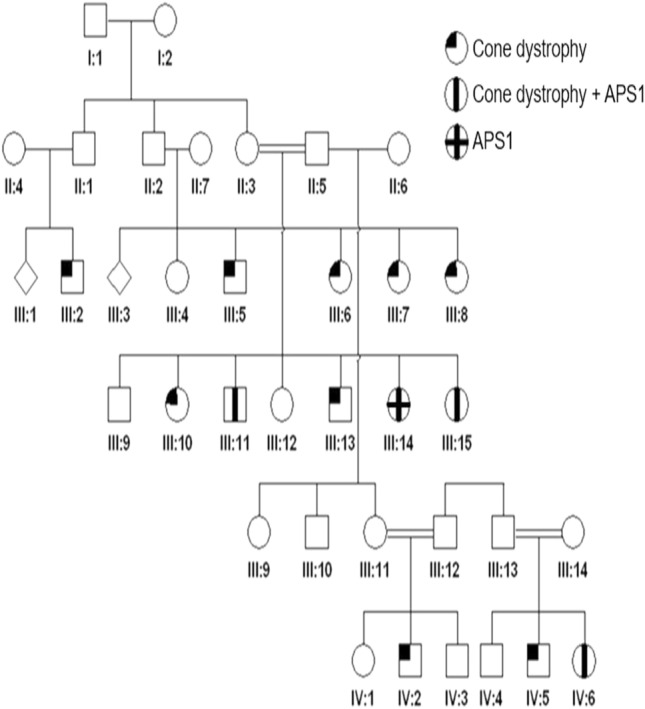
A partial pedigree of the Saudi family showing members with APS1 and/or cone dystrophy.

**Figure 2:**
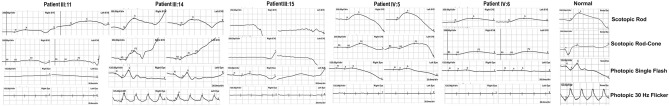
Electroretinogram of 5 family members showing flat photopic and markedly reduced scotopic responses in patient III:11 who is homozygous for the *AIRE* and *PDE6C* variants (the proband). Patient III:14 who is homozygous for the *AIRE* variant and heterozygous for the *PDE6C* variant showed normal scotopic and photopic responses (the proband’s sister). Photopic responses showed Profoundly reduced b wave with delayed implicit time with flat photopic ERG in patient III:15 who is homozygous for the *AIRE* and *PDE6C* variants. Patient IV:5 who is heterozygous for the *AIRE* variant and homozygous for the *PDE6C* variant who had low amplitude and delayed implicit time photopic responses while scotopic ffERG was normal. Patient IV:6 who is homozygous for the *AIRE* and *PDE6C* variants had low amplitude and delayed implicit time photopic responses while scotopic ffERG was normal.

Detailed ophthalmic and systemic history was obtained for 31 family members (Fig. 1). After identification of the proband, 13 family members were brought for genetic testing using targeted variant analysis (Table 1). Ophthalmic examinations were performed on seven family members (Table 2). Figure 2 shows the ffERG which could be obtained from five family members.

**Table 1:** The results of genetic testing in 14 family members of a large Saudi family with Autoimmune polyglandular syndrome and cone dystrophy.

Patient	*AIRE*	*PDE6C*
Patient II:2	Heterozygous	Heterozygous
Patient II:3	Heterozygous	Heterozygous
Patient II:6	Wild type	Wild type
Patient III:4	Wild type	Heterozygous
Patient III:7	Wild type	Heterozygous
Patient III:8	Heterozygous	Heterozygous
Patient III:13	Heterozygous	Heterozygous
Patient III:14	Heterozygous	Heterozygous
Patient III:11 (proband)	Homozygous	Homozygous
Patient III:14 (proband’s sister)	Homozygous	Heterozygous
Pateint III:15	Homozygous	Homozygous
Patient IV:3	Heterozygous	Wild type
Patient IV:5 (proband nephew’s child)	Heterozygous	Homozygous
Patient IV:6	Homozygous	Homozygous

*AIRE* autoimmune regulator gene, *PDE6C* phosphodiesterase 6C gene.

**Table 2:** Ophthalmic examinations of 7 family members from a large Saudi family with autoimmune polyglandular syndrome and cone dystrophy.

Patient	Age	Systemic features	Visual acuity	Cornea	Retina	Fundus autofluorescence	OCT	ERG photopic	ERG scotopic
Patient III:7	5 years	Normal	Fixing and following OU	Clear cornea	Normal	N/A	N/A	Normal	Normal
Patient III:8	3 years	Normal	Fixing and following OU	Clear cornea	Normal	N/A	N/A	Normal	Normal
Patient III:11	25 years	Polyendocrine deficiency	20/300 OU	Autoimmune keratitis	Bull’s eye maculopathy both eyes	Enlarged foveal hypofluorescent area both eyes	Foveal thinning, loss of ellipsoid zone at fovea both eyes	Flat	Reduced, “electro-negative” ERG
Patient III:14	18 years	Polyendocrine deficiency	20/125 OD 20/80 OS	Autoimmune keratitis	Normal	Normal	Normal	Normal	Normal
Patient III15	21 years	Polyendocrine deficiency/Mental retardation	NLP OU	Totally opaque cornea	Not visible	N/A	N/A	Profound reduced b wave and delayed implicit time	Flat
Patient IV:5	5 years	None	20/400 OU	Clear cornea	Dull foveal reflex	Marked foveal hypoautofluorescence	Mild attenuation of ellipsoid zone at fovea both eyes	Low amplitude, delayed implicit time	Normal
Patient IV:6	3 years	Growth retardation	Fixing and following OU	Clear cornea	Dull foveal reflex	N/A	N/A	Low amplitude, delayed implicit time	Normal

*ERG* electroretinogram, *N/A* not available, *OCT* optical coherence tomography, *OD* right eye, *OS* left eye, *OU* both eyes.

Cone dystrophy was documented in three patients, including one presymptomatic patient, who were homozygous for a pathogenic *PDE6C* variant. Three family members were homozygous for both NM_000383.4:c.205_208dupCAGG; p.(Asp70Alafs*148) (Human Gene Mutation Database; HGMD CI991953) in *AIRE* and homozygous for NM_006204.3:c.481-1G>A (HGMD CS2015060) in *PDE6C*, while another two family members were homozygous for either variant. The c.205_208dupCAGG: p.(Asp70Alafs*148) variant in *AIRE* gene causes a frameshift, which alters the proteins amino acid sequence beginning at position 70 and leads to a premature termination codon 148 amino acids downstream therefore completely abolishing the downstream SAND domain and the two Zinc Finger domains. It is predicted to cause a truncated or absent AIRE protein, while the c.481-1G>A variant in *PDE6C* leads to a canonical splicing site (-1) predicted to be deleterious (loss of acceptor site) by in silico tools .Homozygosity for the *PDE6C* variant was associated with cone dystrophy and homozygosity for the *AIRE* variant was associated with APS1. Both variants were described previously^10^. In addition, two family members who were homozygous for the *PDE6C* and *AIRE* variants showed reduced rods function on ERG.

### Patient III:11 (the proband)

**Figure 3:**
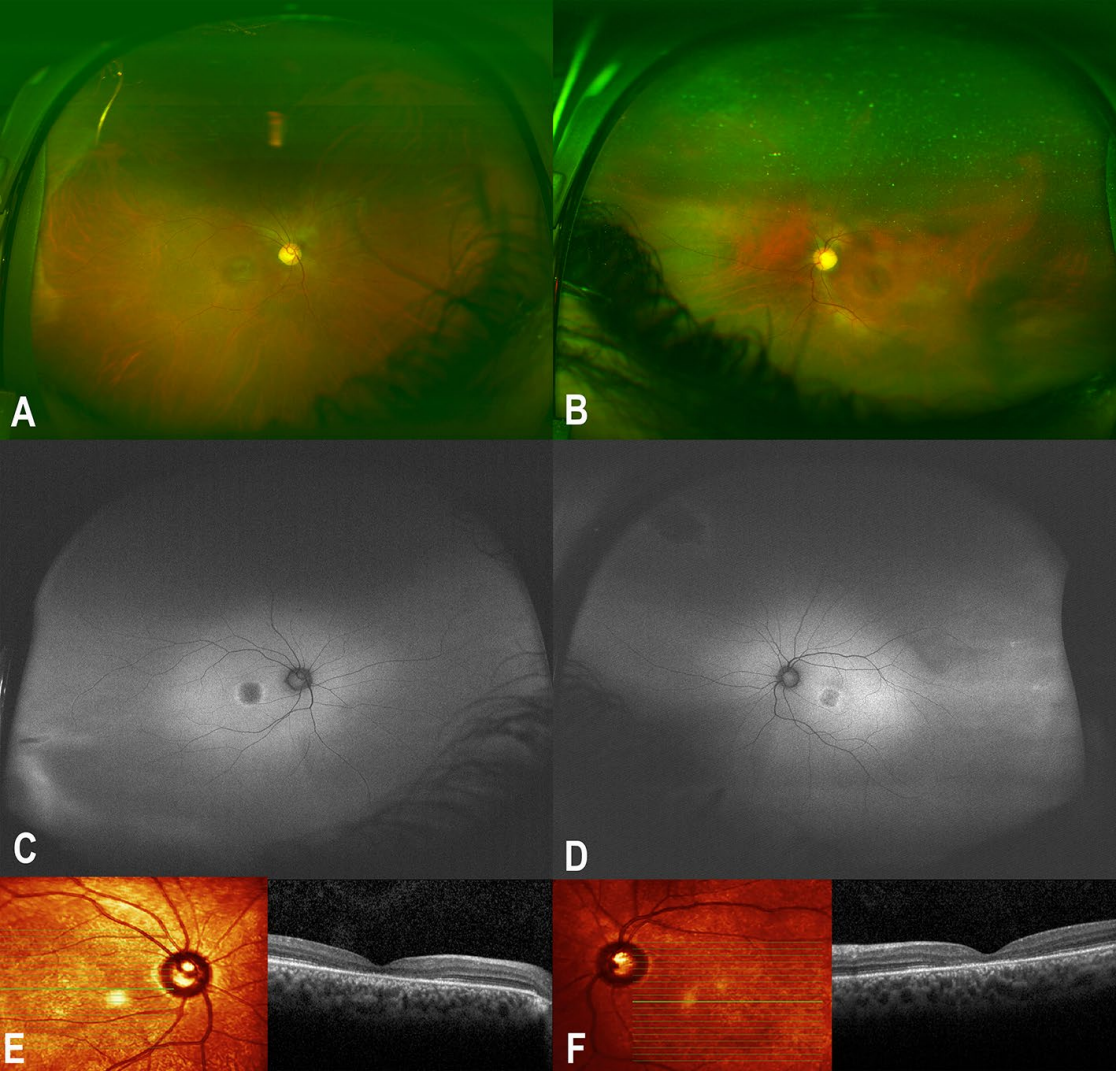
Detailed ophthalmic examination and multimodal imaging in a 25-year-old male patient who is homozygous for the *AIRE* and *PDE6C* variants and manifested both the autoimmune polyglandular syndrome type 1 (APS1) and cone dystrophy (the proband; patient III:11). (**A,B**) Are color fundus photos showing Bull’s eye maculopathy. (**C,D**) Are Fundus Autofluorescence photos showing broad ring of macular hyperautofluorescence and a dull hypoautofluorescent central reflex. (**E,F**) Are Spectral Domain Optical Coherence Tomography (SD-OCT) showing decreased subfoveal thickness and profound loss of the inner segment ellipsoid (ISe) in the foveal area.

A 25-year-old male who is a known case of APS1 presented to the ophthalmology clinic at KKESH with gradual decrease in vision, photophobia, and burning sensation. Systematic review revealed Addison disease, hypothyroidism and hypoparathyroidism. Medication history included oral steroids, thyroid hormone replacement, calcium, and vitamin D. Best-corrected visual acuity (BCVA) was 20/200 in the right eye and 20/300 in the left eye. Corneal examination showed autoimmune keratitis with moderate corneal opacities. Fundus examination showed Bull's eye maculopathy (Fig. 3A,B). Fundus Autofluorescence showed a broad ring of macular hyperautofluorescence and a dull hypoautofluorescent central reflex (Fig. 3C,D). SD-OCT showed decreased subfoveal thickness and profound loss of the inner segment ellipsoid (ISe) in the foveal area (Fig. 3E,F). Goldmann visual field showed loss of peripheral visual field in the right eye and a central scotoma in the left eye. FfERG showed flat photopic and markedly reduced scotopic responses (Fig. 2; Patient III:11). Genetic testing with whole exome sequencing showed two homozygous pathogenic loss of function variants in known disease-related genes: a c.205_208dupCAGG;p.(Asp70Alafs*148) frameshift indel in exon 2 of *AIRE* and a c.481-1G > A canonical splicing variant upstream of exon 2 of *PDE6C*. The patient was treated with topical cyclosporine A 1% as described previously^11^.

### Genetic and ophthalmic examinations of further family members

#### Patient III:14 (proband’s sister)

**Figure 4:**
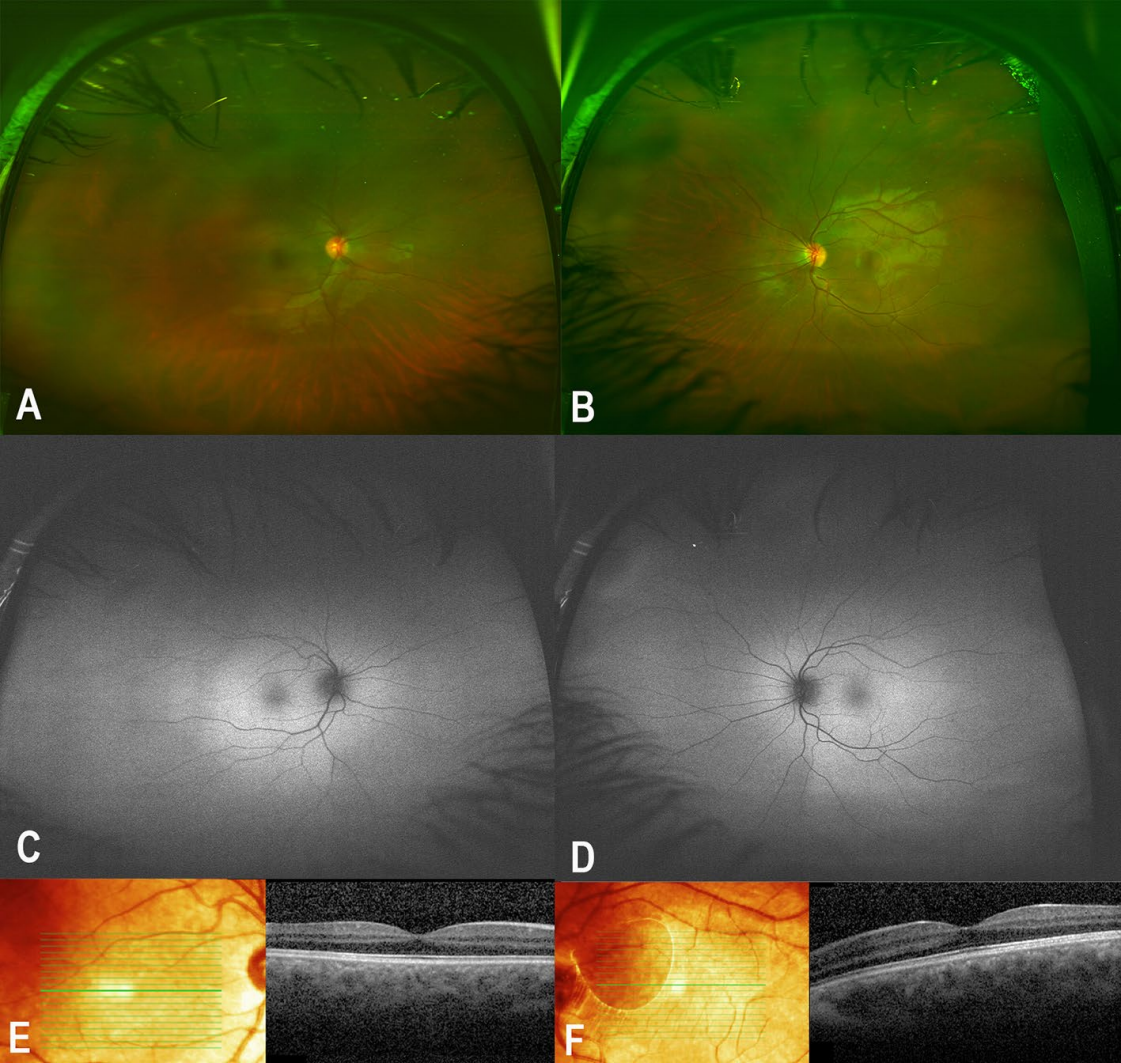
Detailed ophthalmic examination and multimodal imaging in an 18-year-old female patient who is homozygous for the *AIRE* variant and heterozygous for the *PDE6C* variant who manifested APS1 (the proband’s sister, patient III:14). (**A,B**) Are color fundus photos showing normal examination in both eyes. (**C,D**) Are Fundus autofluorescence showing normal autofluorescence in both eyes. (**E,F**) Are SD-OCT showing normal retinal layers in both eyes.

An 18-year-old female who is following with endocrinology as a case of an APS1. Systematic review revealed Addison disease, and hypoparathyroidism for which she was taking cortisol, calcium, and vitamin D. Her BCVA was 20/400 in the right eye and 20/300 in the left eye. Corneal examination showed autoimmune keratitis with moderate corneal opacities. Dilated fundus examination was normal in both eyes (Fig. 4A,B). Fundus autofluorescence showed normal autofluorescence in both eyes (Fig. 4C,D). SD-OCT was normal in both eyes (Fig. 4E,F). FfERG was normal in both eyes (Fig. 2; patient III:14). Genetic testing showed homozygosity and heterozygosity for the familial *AIRE* and *PDEC6C* variants, respectively. The patient was treated with topical cyclosporine A 1% as described previously^11^.

#### Patient IV:5 (proband’s nephew’s child)

**Figure 5:**
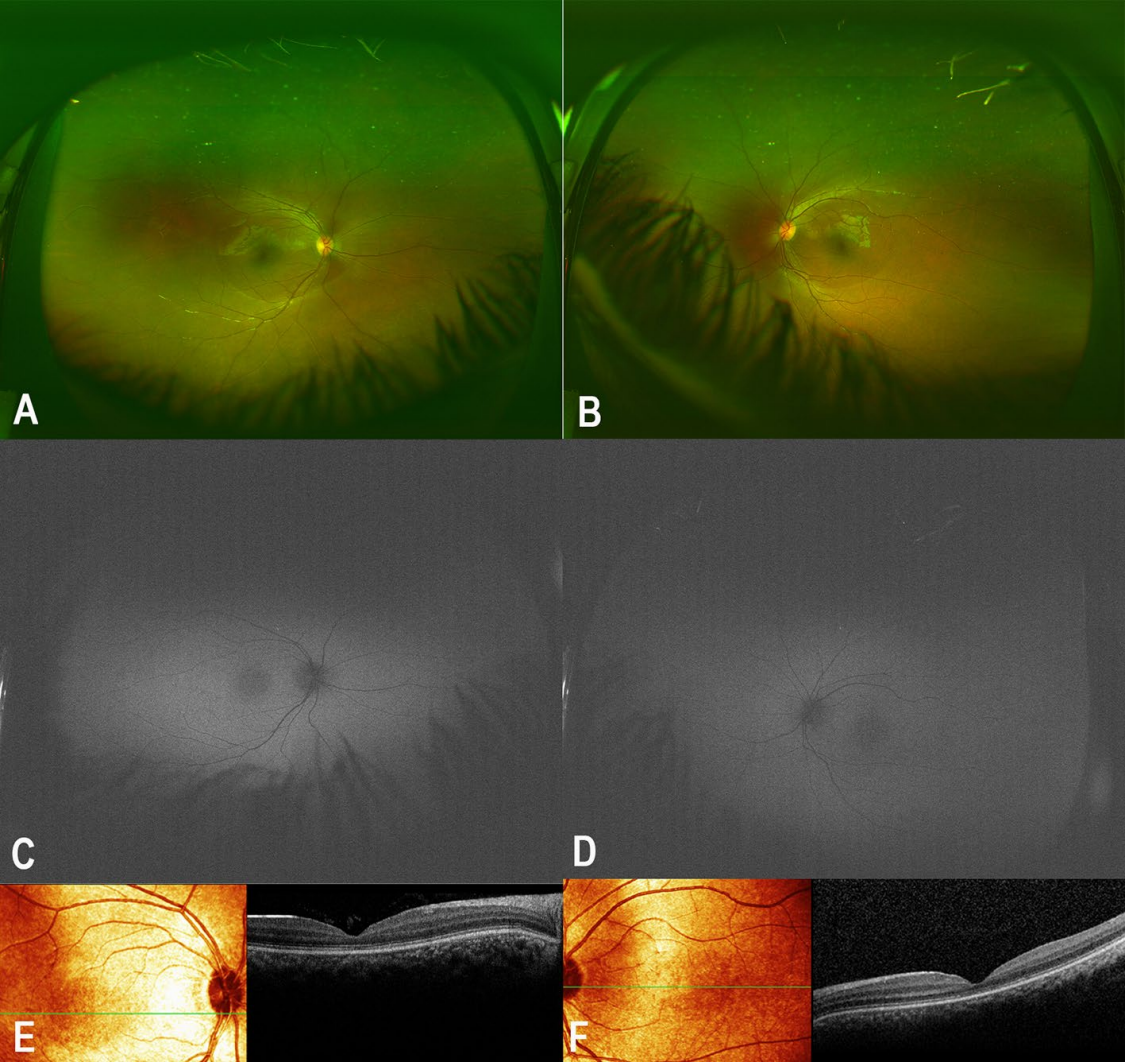
Detailed ophthalmic examination and multimodal imaging in a 5-year-old male who is heterozygous for the *AIRE* and homozygous for the *PDE6C* variant and manifested cone dystrophy (patient IV:5). (**A,B**) Are color fundus photos showing attenuated foveal reflexes in both eyes. (**C,D**) Are Fundus autofluorescence showing Marked foveal hypoautofluorescence in both eyes. (**E,F**) Are SD-OCT showing foveal thinning and Mild attenuation of ellipsoid zone at fovea both eyes.

A 5 year-old boy whose parents denied any systemic or visual problems was examined and showed normal anterior segment, while fundus examination showed attenuated foveal reflexes in both eyes (Fig. 5A,B). Fundus autofluorescence revealed Marked foveal hypoautofluorescence in both eyes (Fig. 5C,D) and SD-OCT revealed foveal thinning and Mild attenuation of ellipsoid zone at fovea both eyes (Fig. 5E,F). Photopic ffERG showed low amplitude and delayed implicit time while scotopic ffERG was normal (Fig. 2; patient IV:5). Genetic testing showed heterozygosity and homozygosity for the familial *AIRE* and *PDE6C* variants, respectively.

## Discussion

In this paper, we describe the ocular findings of a Saudi family with pathogenic variants in *AIRE* and *PDE6C*. The *PDE6C* gene belongs to the Phosphodiesterase 6 (*PDE6*) family. PDE6 is one of 21 enzymes which regulate the intracellular concentration of cyclic nucleotides^12,13^. PDE6 plays an important role in converting light to electrical signals within the neural retina^14^. When the PDE6 enzyme is activated, intracellular cGMP is hydrolyzed, leading to propagation of the visual cycle^15^. While rods have a PDE6 catalytic core composed of a heterodimer of PDE6A and PDE6B subunits which are inhibited by PDE6G, cones have a catalytic homodimer of PDE6C, which is inhibited by PDE6H subunits^16^. This explains why pathogenic variants in *PDE6A* (OMIM #180071), *PDE6B* (OMIM #180072) and *PDE6G* (OMIM #180073) cause predominantly rod-involving retinal dystrophies, whereas pathogenic variants in *PDE6C* and *PDE6H* (OMIM #600827 and #601190) lead to retinal dystrophies which predominantly affect cones^17–19^.

Cone dystrophy was documented in three patients, including one presymptomatic, who were homozygous for the pathogenic *PDE6C* variant. One patient had flat ERG with poor visibility of the fundus due to totally opaque corneas in a patient who was homozygous for the pathogenic variants in both *AIRE* and *PDE6C*. The corneal opacification in this patient could potentially contribute to the flat ERG appearance in this patient. Although peripheral retinitis pigmentosa-like picture was described in APS1, none of our patients had that manifestation^20–22^. Bourgault et al.^22^ found retinitis pigmentosa changes in a series of 5 patients with APS1 with elevated antiretinal antibodies. Additionally, some reports found elevated antiretinal antibodies, raising the possibility of autoimmune retinopathy^23,24^. Histopathological examination of retinal pigmentations in APS1 found focal areas of pigmentation in the inner retina along the retinal blood vessels surrounded by areas of an atrophy of the outer retina^25^. Although these changes were similar to the histopathological findings in retinitis pigmentosa, the exact mechanism which leads to pigment deposition is not known^25,26^.

Among the five cases described by Bourgault et al., three showed macular thinning on examination which were documented by decreased subfoveal thickness and disruption of external limiting membrane and inner segment ellipsoid in SD-OCT^22^. In addition, four out of five patients had reduced or nonrecordable cone function. Limited screening for Leber Congenital Amaurosis (LCA) genes in one of the five patients was negative^22^. Furthermore, macular dystrophic changes were found in one Japanese patient with APS1^27^. All these findings indicate that APS1 patients can manifest cone dystrophy. Cone-rod dystrophies could also be found in other of systemic conditions including endocrinopathies and metabolic diseases, such as the Alstrom syndrome, ciliopathies, fucosidosis, neuronal ceroid lipofuscinosis, infantile phytanic acid storage disease, and methylmalonic aciduria with homocystinuria^28^.

Two patients (patients III:11 and III:15) who are homozygous for c.205_208dupCAGG;p.(Asp70Alafs*148) variant in exon 2 of *AIRE* and homozygous for the c.481-1G>A variant in exon 2 of *PDE6C*, showed rod dysfunction on ERG in addition to the impaired cone responses. The reason for this is not clear. One possibility is that this is caused by an autoimmune retinopathy associated with APS1, similar to what has been suggested previously in the literature^20–22^. The diagnosis of autoimmune retinopathy is confirmed by measurements of the levels of antiretinal antibodies which were not performed in this study. Furthermore, although WES was performed, we cannot completely rule out further intronic variants in other or novel retinopathy genes which could cause this finding. Another less likely possibility is the association with the homozygous *PDE6C* variant^17^. It is unknown whether the c.205_208dupCAGG;p.(Asp70Alafs*148) variant in *AIRE* has affected the rods function and aggravated the retinal phenotype which is affected by the c.481-1G>A variant in *PDE6C*.

Notably, The clinical findings in patient III:11 (the proband) including fundus photos, FAF and SD-OCT did not show signs of auto immune retinopathy including narrowing of the retinal vasculature, retinal pigmentary changes, optic nerve pallor, diffuse posterior pole hyperautofluorescence which spares the fovea and peripheral outer retinal loss (Fig. 3)^29^. Furthermore, the proband’s sister (patient III:14) who is homozygous for the *AIRE* variant and heterozygous for the *PDE6C* variant has got completely normal retinal exam despite having the APS1 disease which further reduces the possibility of auto immune retinopathy in this study.

The increasing use of untargeted genome sequencing assays such as whole exome and whole genome sequencing has greatly increased the visibility of “multilocus” diseases in which the patient’s phenotype is attributed to pathogenic variants in two more genes causing the simultaneous occurrence of more than one monogenic disease in the same patient^30^. Although all combinations of inheritance modes (autosomal, X-linked, recessive and dominant) have been observed, the co-occurrence of two or more independent recessive conditions is particularly prevalent in consanguineous families as shown by us and others^10,31–33^. This should not be surprising since consanguineous couples share a significant proportion of their genomes (e.g. 1/8 in the case of first cousins) so the likelihood of the shared carrier status of more than one pathogenic recessive variant is greatly increased. Indeed, we have recently shown that the classic counseling of consanguineous couples of the 25% recurrence risk of a familial variant overlooks the small but significant recurrence risk for other autosomal recessive variants even when family history is lacking^10^. Therefore, consanguinity may prompt the search of more than one disease-related gene in families in whom the disease phenotype is atypical. This is especially challenging in the absence of family relatives in whom the different components of the phenotype segregate separately.

Limitations of this study include the limited number of family members who were available for ophthalmic examination. In addition, evaluation of this family did not include anti-retinal antibodies testing to explore the possibility of an autoimmune retinopathy which could underlie the rod dysfunction which was observed in 2 of the patients.

In summary we present for the first time the co-occurrence of *PDE6C*-related cone dystrophy with APS1. We suggest that ophthalmologists who encounter unusual presentations of diseases with well-established phenotypic spectrum to consider the possibility of a multilocus disease especially in the setting of consanguinity.

## Data Availability

The datasets generated and/or analyzed during the current study are not publicly available due to limitations of ethical approval involving the patient data and anonymity but are available from the corresponding author on reasonable request.

## References

[1] Ahonen P, Myllarniemi S, Sipila I, Perheentupa J (1990). Clinical variation of autoimmune polyendocrinopathy-candidiasis-ectodermal dystrophy (APECED) in a series of 68 patients. N. Engl. J. Med..

[2] Nagamine K, Peterson P, Scott HS (1997). Positional cloning of the APECED gene. Nat. Genet..

[3] Björses P, Pelto-Huikko M, Kaukonen J, Aaltonen J, Peltonen L, Ulmanen I (1999). Localization of the APECED protein in distinct nuclear structures. Hum. Mol. Genet..

[4] Björses P, Halonen M, Palvimo JJ (2000). Mutations in the AIRE gene: Effects on subcellular location and transactivation function of the autoimmune polyendocrinopathy-candidiasis-ectodermal dystrophy protein. Am. J. Hum. Genet..

[5] Couturier A, Brézin AP (2016). Ocular manifestations of autoimmune polyendocrinopathy syndrome type 1. Curr. Opin. Ophthalmol..

[6] Yeh S, de Paiva CS, Hwang CS, Trinca K, Lingappan A, Rafati JK, Farley WJ, Li DQ, Pflugfelder SC (2009). Spontaneous T cell mediated keratoconjunctivitis in Aire-deficient mice. Br. J. Ophthalmol..

[7] Li S, Nikulina K, DeVoss J, Wu AJ, Strauss EC, Anderson MS, McNamara NA (2008). Small proline-rich protein 1B (SPRR1B) is a biomarker for squamous metaplasia in dry eye disease. Investig. Ophthalmol. Vis. Sci..

[8] DeVoss J (2007). Spontaneous autoimmunity prevented by thymic expression of a single self-antigen. J. Exp. Med..

[9] Magliyah MS, AlSulaiman SM, Schatz P, Nowilaty SR (2021). Evolution of macular hole in enhanced S-cone syndrome. Doc. Ophthalmol..

[10] AlAbdi L, Alrashseed S, Alsulaiman A (2021). Residual risk for additional recessive diseases in consanguineous couples. Genet. Med..

[11] AlAbbasi O, Magliyah MS, Ahad M (2021). Long term keratits treatment with topical cyclosporin a in autoimmune polyglandular syndrome type 1. Am. J. Ophthalmol. Case Rep..

[12] Bender AT, Beavo JA (2006). Cyclic nucleotide phosphodiesterases: Molecular regulation to clinical use. Pharmacol. Rev..

[13] Lagman D, Franzén IE, Eggert J, Larhammar D, Abalo XM (2016). Evolution and expression of the phosphodiesterase 6 genes unveils vertebrate novelty to control photosensitivity. BMC Evol. Biol..

[14] Arshavsky VY, Lamb TD, Pugh EN (2002). G proteins and phototransduction. Annu. Rev. Physiol..

[15] Cote RH (2004). Characteristics of photoreceptor PDE (PDE6): Similarities and differences to PDE5. Int. J. Impot. Res..

[16] Gopalakrishna KN, Boyd K, Artemyev NO (2017). Mechanisms of mutant PDE6 proteins underlying retinal diseases. Cell Signal..

[17] Daich Varela M, Ullah E, Yousaf S, Brooks BP, Hufnagel RB, Huryn LA (2020). PDE6C: Novel mutations, atypical phenotype, and differences among children and adults. Investig. Ophthalmol. Vis. Sci..

[18] Thiadens AA, den Hollander AI, Roosing S (2009). Homozygosity mapping reveals PDE6C mutations in patients with early-onset cone photoreceptor disorders. Am. J. Hum. Genet..

[19] Kohl S, Coppieters F, Meire F (2012). A nonsense mutation in PDE6H causes autosomal-recessive incomplete achromatopsia. Am. J. Hum. Genet..

[20] Gass JD (1962). The syndrome of keratoconjunctivitis, superficial moniliasis, idiopathic hypoparathyroidism, and Addison’s disease. Am. J. Ophthalmol..

[21] Orlova EM, Bukina AM, Kuznetsova ES (2010). Autoimmune polyglandular syndrome type 1 in Russian patients: Clinical variants and autoimmune regulator mutations. Horm. Res. Paediatr..

[22] Bourgault S, Baril C, Vincent A, Héon E, Ali A, MacDonald I, Lueder GT, Colleaux KM, Laliberté I (2015). Retinal degeneration in autoimmune polyglandular syndrome type 1: A case series. Br. J. Ophthalmol..

[23] Breunig A, Lee MS, Miller BS, Binstadt BA, Anderson MS, Montezuma S (2013). Autoimmune retinopathy in a patient with autoimmune polyendocrine syndrome type I. Ocul. Immunol. Inflamm..

[24] Wood LW, Jampol LM, Daily MJ (1991). Retinal and optic nerve manifestations of autoimmune polyendocrinopathy-candidiasis-ectodermal dystrophy. Arch. Ophthalmol..

[25] Culp CJ, Pappas CM, Toso M, Qu P, Mamalis N, Hageman GS (2022). Clinical, histological and genetic findings in a donor with a clinical history of type 1 autoimmune polyendocrinopathy syndrome. Am. J. Ophthalmol. Case Rep..

[26] Milam AH, Li ZY, Fariss RN (1998). Histopathology of the human retina in retinitis pigmentosa. Prog. Retin. Eye Res..

[27] Haruta M, Tsuji T, Yoshida S (2021). Ultra-widefield OCT in retinopathy of autoimmune polyendocrine syndrome type 1. Ophthalmol. Retina.

[28] Traboulsi EI (2012). Genetic Diseases of the Eye.

[29] Canamary AM, Takahashi WY, Sallum JMF (2018). Autoimmune retinopathy: A review. Int. J. Retina Vitreous.

[30] Posey JE, Harel T, Liu P (2017). Resolution of disease phenotypes resulting from multilocus genomic variation. N. Engl. J Med..

[31] Mitani T, Isikay S, Gezdirici A (2021). High prevalence of multilocus pathogenic variation in neurodevelopmental disorders in the Turkish population. Am. J. Hum. Genet..

[32] Monies, D. *et al*. Lessons learned from large-scale, first-tier clinical exome sequencing in a highly consanguineous population. *Am. J. Hum. Genet*. 104(6), 1182–1201. 10.1016/j.ajhg.2019.04.011 (2019). Erratum in: *Am. J. Hum. Genet.* 105(4), 879 (2019). 10.1016/j.ajhg.2019.04.011 PMC6562004 31130284

[33] Monies D, Abouelhoda M, AlSayed M (2017). The landscape of genetic diseases in Saudi Arabia based on the first 1000 diagnostic panels and exomes. Hum. Genet..

